# Quantifying User Satisfaction: Weighted Metric Approach for Evaluating Deep Learning-Based Thigh MRI Segmentations

**DOI:** 10.3390/diagnostics16152425

**Published:** 2026-07-31

**Authors:** Falko Ensle, Ilker Özgür Koska, Nina Derron, Cagan Koska, Ulf Bach, Philipp Nikolaus Maintz, Marta Porta-Vilarό, Jonas Kroschke, Philipp Gerber, Roman Guggenberger

**Affiliations:** 1 Diagnostic and Interventional Radiology, University Hospital of Zurich, 8091 Zurich, Switzerland; 2 Department of Pediatric Radiology, Acıbadem Kent Hospital, 35620 Izmir, Türkiye; 3 Biomedical Technologies Department, Engineering Faculty, Dokuz Eylül University, 35390 Izmir, Türkiye; 4 Department of Endocrinology, Diabetology and Clinical Nutrition, University Hospital of Zurich, 8091 Zurich, Switzerland; 5 Department of Electrical Electronical, Engineering Faculty, Yaşar University, 35100 Izmir, Türkiye; 6 Department of Radiology, Hospital Clínic de Barcelona, 08036 Barcelona, Spain; 7 Department of Radiology and Nuclear Medicine, Cantonal Hospital Winterthur, University of Zurich, 8091 Zurich, Switzerland

**Keywords:** MRI, segmentation, deep learning, convolutional neural network, thigh, quantitative

## Abstract

**Background:** The aim of this paper is to develop a clinically useful benchmark signature of deep learning (DL)-based MRI segmentations and objectively predict user satisfaction based on a weighted combination of multiple performance metrics. **Methods:** This post hoc analysis of a prospective study analyzed MRI data from 68 patients (29.4 ± 5.6 years; 45% female) acquired during a randomized clinical trial. Fat fraction maps of axial Dixon MRI were used to segment six classes of the thigh. Two radiologists qualitatively scored DL-based segmentations using a 5-point Likert scale. A hybrid deep learning model (MobileNetV2 and DINOv2) was developed to predict Likert scores directly from MRI slices and segmentation maps. Additionally, a weighted combination of quantitative metrics (Dice score, Hausdorff distance, Jaccard index) was determined to best correlate with the Likert scores. Statistical analyses included Cohen’s kappa, Spearman correlation, and regression model performance (MAE, RMSE, ROC_AUC). **Results:** Interreader agreement for Likert scores was near-perfect (κ = 0.82). The DINOv2 model achieved lower MAE (0.2343) and RMSE (0.1129) than MobileNetV2 (0.3488 and 0.431, respectively) in predicting Likert scores. The weighted metric combination model, using Lasso regression, predicted Likert = 5 with 84.1% accuracy, 100% recall, and ROC_AUC of 0.824. Individual metrics showed weak-to-moderate correlation with Likert scores, with Dice and Jaccard indices for extensor intramuscular fat exhibiting the highest correlation (r = 0.37 and r = 0.34, respectively). **Conclusions:** The proposed DL-based model accurately predicted radiologist-assigned Likert five scores, while the weighted metric combination model aligned closely with user satisfaction, offering a practical alternative to manual validation for clinical adoption of automated segmentation tools.

## 1. Introduction

Accurate segmentation of musculoskeletal structures in magnetic resonance imaging (MRI) is becoming increasingly relevant for diagnosis, personalized treatment planning, and longitudinal monitoring of disorders such as sarcopenia, osteoarthritis or metabolic syndromes [1,2,3,4,5]. The recent advent of deep learning (DL)-based techniques has enabled to largely automate the previously tedious segmentation process, supporting its large-scale application, with continuous advancements toward more robust and precise algorithms [6,7,8]. However, clinical adoption of these algorithms often remains limited and is relatively slow, partly due to a lack of clinically reasonable metrics of their performance that could guide the user regarding their utility for real-world scenarios/tasks [9]. Most DL-based segmentation models are evaluated based solely on quantitative measures, such as Dice scores, Hausdorff distances or Jaccard indices. While these metrics objectively reflect the technical performance of the algorithm, they may fail to uncover small but clinically meaningful errors, such as misclassification of fat-muscle interfaces [10]. This mismatch between automated metrics and user satisfaction represents a major barrier to the clinical adoption of automated segmentation tools, requiring time-consuming manual reviews to ensure reliability for specific applications and needs. The addition of qualitative metrics, e.g., based on Likert scales, could provide a more clinically useful and user-centered assessment of segmentation performance. Prior research has primarily focused on improving technical aspects of segmentation algorithms, largely neglecting the potential gap between technical performance and clinical utility/needs [11].

In this study, we aim to address this gap by developing two complementary methods to objectively predict user satisfaction with automated segmentations of Dixon thigh MRI. We hypothesize that (1) a DL-based model can accurately predict radiologist-assigned Likert scores, effectively identifying insufficient segmentations before clinical review, and (2) a weighted combination of quantitative metrics will correlate more closely with user satisfaction than individual metrics alone, offering a practical alternative to manual validation.

## 2. Methods

### 2.1. Study Design

This post hoc analysis of MRI data was based on a randomized clinical trial with previously published findings, which compared metabolic effects of intermittent fasting protocols [12]. The trial included healthy, non-obese participants that were randomized into three intervention groups. The groups were comparable regarding age, sex distribution, body-mass index and biochemical parameters of glucose and lipid metabolism. At baseline and follow-up after a month-long study intervention, participants underwent whole-body MRI to assess body composition changes. As an adjunct to this trial, an axial multi-echo Dixon gradient echo volumetric interpolated breath-hold examination (VIBE) sequence covering the mid-thigh bilaterally was prospectively acquired under an IRB-approved protocol in 68 patients at baseline and follow-up. The effective unit of analysis of this study was the MRI from 68 subjects, yielding a two-dimensional (2D) axial slice derived from bilateral thighs (×2), two imaging time points (×136), and multiple slices per leg (×30), yielding 8160 slice level samples. The MR sequences were acquired on one of the two 3 Tesla clinical scanners (MAGNETOM Vida fit, Siemens Healthineers AG, Erlangen, Germany) using a 36-channel posterior array coil with the following parameters: slice thickness, 5 mm; image resolution, 0.8 × 0.8 × 3.0 mm^3^; matrix size, 448 × 364 (224 × 182); time to repetition, 19.7 ms; echo time, 1.04 ms; bandwidth, 1488 Hz/px; flip angle, 3°; and acquisition time, 1:17 min.

### 2.2. Segmentation Methodology and Grading

For each leg, manual segmentation of the fat fraction (FF) map was performed by a radiology fellow with 5 years of experience using ITK-SNAP (http://www.itksnap.org, accessed on 1 August 2025) on three slices, one at mid-level of the thigh and one each at the proximal and distal locations with a gap of 5 slices from mid-level, respectively. In total, 6 classes were segmented: muscle and intermuscular fat of the extensor and flexor compartments, respectively, in addition to perimuscular fat (deep to the fascia lata) and subcutaneous fat. Segmentation masks were saved in DICOM format and used as a training and validation dataset for designing an algorithm based on a deep learning U-Net architecture for fully automated segmentation of entire 2D image stack of the thigh MRI [13,14]. This model employed a symmetrical U-Net design with a contracting path (encoder) of 5 convolutional blocks (each with two 3 × 3 convolutions and 2 × 2 max-pooling) and an expanding path (decoder) of 4 blocks (bilinear upsampling followed by two 3 × 3 convolutions). Skip connections concatenated encoder feature maps to the decoder to preserve spatial resolution. Batch normalization stabilized training after convolutional layers, while rectified linear units activations were utilized across all hidden layers to handle non-linearities. For output mapping, the final layer used a 1 × 1 convolution mapped to an activation layer to output pixel-wise probability masks for each of the target tissues. The model operated as a 2D network that processes the MRI dataset slice-by-slice, rather than using a 3D volumetric convolution matrix. By relying on standard 2D convolutions rather than 3D blocks, the model required significantly fewer learnable parameters. This allowed it to train robustly on a relatively small, highly curated dataset of 20 fully, manually segmented whole-body MRI scans. Similarly, in the thigh dataset the slices from these 20 patients were used for training. Performance metrics of the model were Dice score 0.789 (95% CI: 0.783–0.795), intersection over union 0.6855 (95% CI: 0.679–0.691) and Hausdorff distance 46.9487 (95% CI: 43.945–49.952).

A board-certified radiologist with 7 years of experience and a radiology resident in-training with 4 years of experience qualitatively scored the DL-based segmentation masks based on a 5-point Likert scale: 5-perfect match, which can be used as is, where no corrections are needed (>95% match); 4-good match, which needs minor corrections (ca. 80–95% match); 3-acceptable match, which needs moderate correction (ca. 60–79% match); 2-fair match, which needs extensive correction (ca. 40–59%); and 1-poor match (less than 40%). The radiologists performed the gradings independently, and they were blinded to each other‘s scores and to the quantitative metrics.

### 2.3. Likert Score Estimation Model

A model was developed that estimates the radiologist-assigned Likert score by stacking the MRI slice and segmentation prediction, running it through a feature extractor. To develop a robust and generalizable imaging-based regression framework, a hybrid deep learning approach was implemented, combining convolutional and transformer-based architectures. The model was designed to predict a radiological assessment score directly from MR image inputs and their derived feature maps. Two different feature extractors were employed as backbone networks: MobileNetV2 and DINOv2.

MobileNetV2 was chosen as the representative lightweight CNN that maintains strong feature extraction capability while substantially reducing computational complexity [15]. Its use of depth-wise separable convolutions and inverted residual blocks allows efficient parameter utilization and stable training even with relatively limited medical imaging datasets—a common constraint in clinical research. These properties make MobileNetV2 particularly suitable for medical applications requiring scalable, resource-efficient deployment.

In parallel, DINOv2, a self-supervised vision transformer (ViT) trained on a large-scale corpus of natural images, was incorporated to leverage its superior capacity for capturing global contextual relationships across the image [16]. Unlike purely convolutional models, transformer-based architectures such as DINOv2 employ attention mechanisms that can model long-range dependencies and subtle spatial interactions, which are often critical in radiologic interpretation. We used the ViT-S/14 variant of DINOv2 (Facebook/dinov2-small checkpoint) from the Hugging Face Transformers library, with self-supervised pretrained weights provided by Meta AI. The backbone was kept frozen, and a downstream prediction head was trained on its extracted features in a linear probing setup to assess representation quality rather than fine-tune the backbone.

The high-dimensional embeddings produced by each backbone were subsequently passed through a fully connected regression head, consisting of sequential dense layers with rectified linear unit activation functions. This head performed the final mapping from image-derived representations to a discrete output variable (a Likert-scale radiologic score). Model optimization was conducted using the Adam optimizer and mean squared error loss.

**Figure 1:**
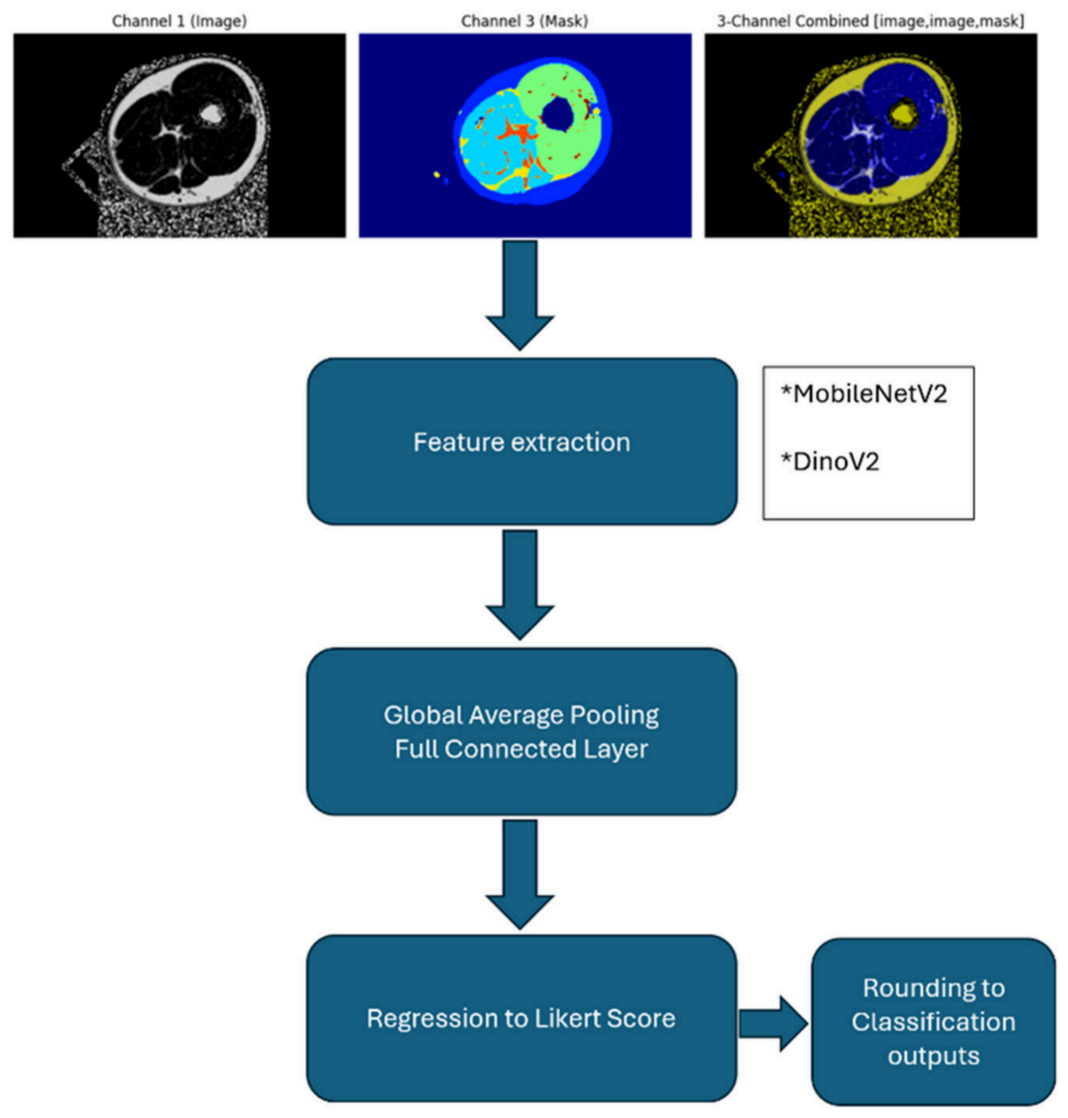
Flowchart demonstrating the development of a Likert score estimation model, to predict a radiological assessment score directly from MR image inputs and their derived feature maps. MRI slice and segmentation predictions were stacked and run through a feature extractor based on a hybrid deep learning approach, i.e., MobileNetV2 and DINOv2 (indicated by *). The high-dimensional embeddings produced by each backbone were subsequently passed through a fully connected regression head, consisting of sequential dense layers with ReLU activation functions. This head performed the final mapping from image-derived representations to a continuous output variable (a Likert-scale radiologic score). Model optimization was conducted using the Adam optimizer and mean squared error loss.

To use MobileNetV2 and DinoV2 as pretrained feature extractors, the input was generated as three channels: two axial images and one segmentation map. Each axial MRI image was repeated twice and stacked with the corresponding segmentation prediction. Each MRI image and segmentation map was first min-max normalized to a range of 0–1, then the imageNET mean was extracted, and then shifted to a range of 0–255. Two separate images were obtained from each leg, left and right, resulting in a total of 8160 (136 × 30 × 2) samples from 30 slices by 68 patients at two time points. Each sample was resized to 224 × 224 × 3. Twenty percent of the samples were allocated as the test set, with separation performed at the patient level. This prevented data leakage by ensuring that any slice from any leg of any patient at any time point was included in either the train or test set. DinoV2 embeddings were 384× [7 × 7], and MobileNetV2 embeddings were 1280× [5 × 5]. Global average pooling was applied to obtain vectors with lengths of 384 and 1280 dim, respectively. Regression was then performed by adding fully connected layers of 128 and 256 dim, respectively. Adam was used as the optimizer; mean squared error (MSE) was used as the loss function, and mean absolute error (MAE) and R2 were used as metrics. Regression results were converted into classification results by rounding to the nearest integer. Due to the highly skewed distribution of the Likert scores in our dataset (where scores 1 and 2 were highly underrepresented, while scores 4 and 5 formed the majority), an ordinal classification framework would suffer from severe class imbalance and optimization instability. Therefore, we formulated the task as a continuous regression problem across the 1–5 range followed by a post hoc rounding step, which improves model robustness and preserves global target gradients. A flowchart demonstrating the development of the Likert score estimation model is illustrated in Figure 1.

### 2.4. Weighted Metric Combination Model

A second model was developed to find an optimal weighted combination of metrics (Dice Score, Hausdorff distance, Jaccard indices) to reflect user satisfaction. The same allocation to training and validation sets as for the Likert score estimation model was applied. Dice score, Hausdorff distance, and Jaccard index for each of the 6 classes for each thigh was calculated using ground truth annotations and the segmentation predictions. The six classes corresponded to the following structures: intermuscular fat, c1; extensor intramuscular fat, c2; subcutaneous fat, c3; extensor muscles, c4; flexor intramuscular fat, c5; and flexor muscles, c6. The resulting 21 features (6 classes × 3 metrics plus the mean of each metric) were input to a regression network. Spearman correlation analysis was applied between these metrics and the Likert scores, in order to assess how each of these metrics align with the Likert scores. Then a linear regression model was built using these metrics as input and predicted the Likert score to discover a relevant weighted combination which aligns better with the Likert scores. The weights of this linear regression model were used as a metric signature to build a logistic regression model to predict if Likert = 5 with this model. Then we explored whether adding sparsity to our regression model would better align with the Likert. To add sparsity, we leveraged Lasso regression to explore the most relevant metrics for prediction of Likert scores. Lasso regression adds L1 distance to the loss function to penalize high weights. Therefore some coefficients become zero, and thereby some features are eliminated.

### 2.5. Statistical Analysis

Interreader agreement between the two radiologists assigning the Likert scores was assessed using quadratic weighted Cohen’s kappa (*κ*) statistics. Degree of agreement was considered poor (*κ* < 0), slight (*κ* = 0.00–0.20), fair (*κ* = 0.21–0.40), moderate (*κ* = 0.41–0.60), substantial (*κ* = 0.61–0.80), or near-perfect (*κ* = 0.81–1.00). Additionally, Spearman’s rank correlation coefficient was used to assess the correlation of the Likert scores. Level of correlation was interpreted as follows: negligible (0.00–0.20), weak (0.21–0.40), moderate (0.41–0.60), strong (0.61–0.80), and very strong (0.81–1.00) [17].

For the Likert score estimation model, performance was assessed using mean absolute error (MAE) and root mean square error (RMSE) to quantify the agreement between predicted and manually assigned Likert scores. These metrics were calculated for both the MobileNetV2 and DINOv2 feature extractors.

For the weighted metric combination model, Dice score, Hausdorff distance, and Jaccard index were calculated for each of the six segmented classes in each thigh to compare ground truth annotations and segmentation predictions. Spearman correlation analysis was applied to evaluate the relationship between these quantitative performance metrics and the radiologist-assigned Likert scores.

The performance of the linear regression model and Lasso regression model was evaluated using accuracy, precision, recall, F1-score, and the area under the receiver operating characteristic curve (ROC_AUC). Additionally, a calibration analysis for the logistic and Lasso regression models was performed.

Statistical significance was set to *p* < 0.05. Statistical analyses were conducted in Python using the SciPy library (version 1.14.1).

## 3. Results

Demographics of the included subjects are demonstrated in Table 1. Interreader agreement between the radiologists assigning the Likert scores was near-perfect (*κ* = 0.82), with very strong correlation of the Likert scores (r_s_ = 0.82).

### 3.1. Likert Score Estimation Model

Classification results of MobileNetV2 and DINOv2 feature extractors are summarized in Table 2. MobileNetV2 demonstrated a MAE of 0.2781 and RMSE of 0.3647 for the training set, while the test set demonstrated a MAE of 0.3488 and RMSE of 0.4314. The regression metrics of DINOv2 indicated a MAE of 0.2552 and RMSE of 0.1381 for training set, while the test demonstrated a MAE of 0.2343 and RMSE of 0.1129.

### 3.2. Weighted Metric Combination Model

**Figure 2:**
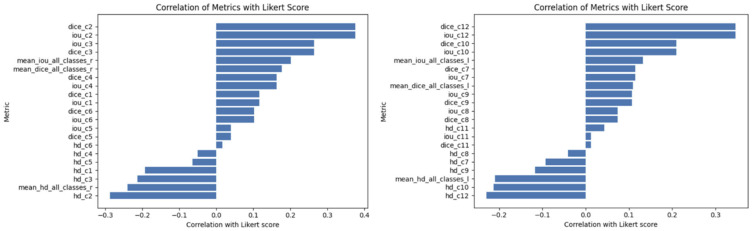
Bar plots demonstrating correlation of metrics with the radiologist-assigned Likert score. Hd, hausdorf distance; IOU, intersection over union.

Overview of metrics correlation with Likert scores is shown in Figure 2. Dice and Jaccard index (i.e., intersection over union (IOU)) score of class 2 (extensor intramuscular fat) was the most correlated metric with r = 0.37, followed by that of class 3 (subcutaneous fat) with r = 0.26 for the right thigh and class 12 (extensor intramuscular fat) with r = 0.34, and class 10 (extensor muscle) with r = 0.2 for the left thigh (Table 3).

**Figure 3:**
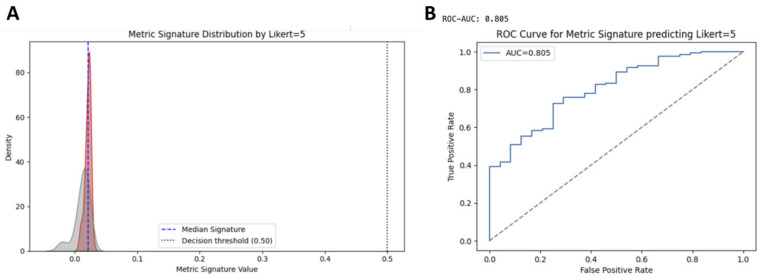
Kernel density estimation plot (**A**) of the logistic regression model showing moderate separation of Likert = 5 from the non-Likert = 5 group. Receiver operating curve (ROC) (**B**) demonstrates an area under the curve (AUC) of 0.805 for metric signature predicting Likert = 5. Selected features and weights for the logistic and Lasso regression models are summarized in Table 3.

With the linear regression model, we obtained MAE: 0.204 and R^2^: 0.181. The logistic regression model based on the weights of the linear regression model predicted Likert = 5 with the following performance metrics: accuracy, 0.846 (95% CI: 0.789–0.903); precision, 0.846 (95% CI: 0.789–0.903); recall, 1.000 (95% CI: 1.000–1.000); F1-score, 0.917 (95% CI: 0.874–0.960); and ROC_AUC, 0.805 (95% CI: 0.743–0.867). Figure 3 illustrates the performance of the logistic regression model.

**Figure 4:**
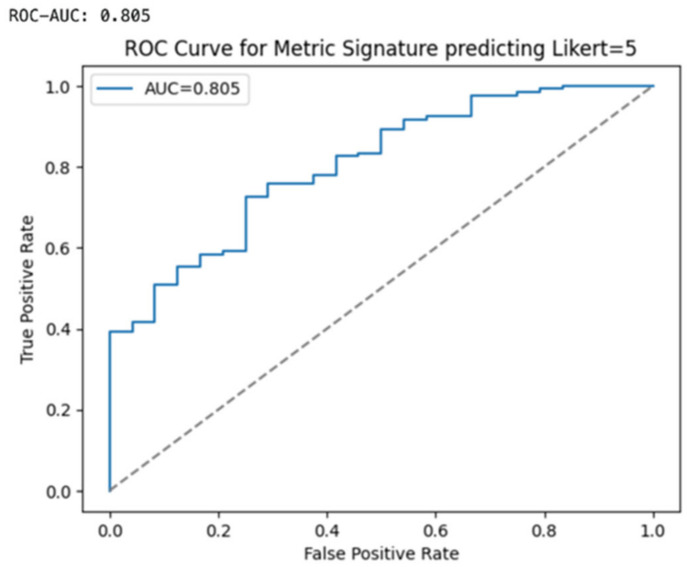
Receiver operating curve (ROC) of the Lasso metric signature model demonstrates an area under the curve (AUC) of 0.805 for metric signature predicting Likert = 5.

**Figure 5:**
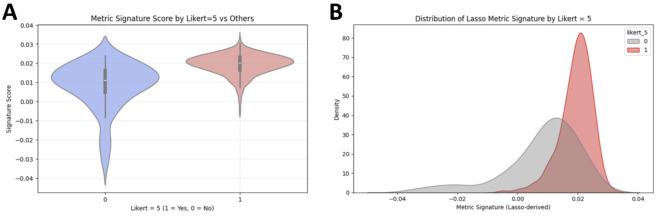
Violin plot with box plots (**A**) and kernel density estimate graph (**B**) demonstrate separation of Likert = 5 vs. non = 5 by Lasso metric signature. The mean metric signature for non-5 group was 0.1, whereas it was 0.2 for Likert = 5 group. There was no Likert = 5 with metric signatures less than −0.1 and only minority of Likert = 5 group had a metric signature between 0.1 and −0.1.

The Lasso regression model predicted Likert = 5 with the following performance metrics: accuracy, 0.841 (95% CI: 0.784–0.898); precision, 0.841 (95% CI: 0.784–0.898); recall, 1.000 (95% CI: 1.000–1.000); F1-score, 0.913 (95% CI: 0.869–0.957); and ROC_AUC, 0.824 (95% CI: 0.764–0.884) (Figure 4 and Figure 5). Based on the calibration analysis, the Brier score was 0.1143 for the logistic and 0.1165 for the Lasso regression model, indicating well calibrated models for this task.

## 4. Discussion

In this study, we proposed a DL-based model that estimates radiologist-assigned Likert five scores directly from Dixon thigh MRI slices and their corresponding segmentations. Second, we investigated whether traditional segmentation metrics (i.e., Dice, Hausdorff, Jaccard) can be weighted and combined to better reflect user satisfaction. Our DL-based model demonstrated accurate predictions of the highest radiologist-assigned Likert five scores to Dixon fat fraction map segmentations of the thigh. While the individual quantitative segmentation metrics showed only weak-to-moderate correlations with Likert scores, the model based on a weighted combination of quantitative metrics correlated closely with Likert five scores. The Likert estimation model can serve as a quality control layer for MRI segmentation algorithms, filtering out insufficient segmentations (Likert scores ≤ 4) and averting time-consuming manual reviews. The weighted combination model allows the assessment of user satisfaction, instead of relying solely on individual metrics for performance assessment of segmentation algorithms. The main contribution of the model is accurate prediction of Likert five scores, which has the potential to be used as a quality control step for segmentations before they are even presented to the observer. The variance that the regression model fails to capture lies almost entirely within the intermediate scores (e.g., distinguishing a Likert = 2 from a Likert = 3). In thigh MR parsing, these intermediate differences are clinically irrelevant since both lead to the same outcome (rejection/re-editing) and are highly prone to inter-observer subjectivity among radiologists. In a real-world clinical workflow, the proposed framework could be implemented via an automated quality control pipeline. In the first step, the model would automatically predict Likert scores for each segmentation output as soon as it is generated, flagging low-quality results (such as Likert <4) for manual review. This could be embedded in the image viewer as a background validation layer. In the second step, the pipeline could provide actionable feedback for the flagged cases, highlighting which anatomical regions or errors potentially require correction.

In the last decade, DL-based muscle and fat segmentation of MRI has demonstrated increasing potential for clinical applications in body composition profiling, suggesting similar results compared to manual segmentations [18,19]. Numerous prior studies in recent years have demonstrated technically accurate DL-based segmentation of musculoskeletal tissue compartments with a slice-based design [20]. However, individual metrics for the performance assessment of machine learning models often do not align well with user satisfaction and do not take into account specific clinical needs. Particularly in the thigh, segmentation can be challenging due to overlap of intensity values in different tissues such as intermuscular fat [21]. This has resulted in reports that segmentation algorithms demonstrate limited usability outside of research environments, with noted discrepancy between published performance and clinical implementation [22,23]. This discrepancy has been described in previous works [24,25], emphasizing that standard segmentation metrics may not correlate with clinical relevance. For instance, while commonly used as an outcome in segmentation studies, the Dice index has shortcomings such as not accounting for spatial features and not distinguishing between types of segmentation errors [26]. Selecting appropriate evaluation metrics for medical image segmentation among the various existing options is not a trivial task [9]. One approach to this challenge has been to include a consistent set of metrics as part of an efficient evaluation tool, to obtain a comprehensive overview [27]. However, such an approach does not account for different weightings of metrics that may hold varying levels of clinical relevance or importance depending on the specific application and user needs. Therefore, our proposed model represents a tailor-made, weighted combination of multiple performance metrics, based on qualitative gradings by radiologists. To our knowledge, such a model has not yet been demonstrated in MRI segmentation.

Based on Dixon thigh MRI data, a regression framework was developed using a hybrid deep learning approach and combining quantitative performance metrics. The integration of DINOv2, a self-supervised vision transformer (ViT) pretrained on large-scale datasets, represents a novel approach in addressing the limitations of traditional CNNs in medical imaging analysis [16]. Classical CNNs often suffer from single-task training constraints and limited representative capacity, requiring extensive labeled datasets for each new application [6]. This limits application into clinical practice, as generating medical annotations is time-consuming and requires expert input [28]. In contrast, DINOv2 is a versatile backbone model trained on extensive datasets for a range of applications, such as image classification, retrieval, depth estimation, and semantic segmentation. It leverages self-supervised learning and attention layers, producing all-purpose visual features that work across image distributions and tasks without fine-tuning. This capability is particularly valuable in MRI, where anatomical structures are depicted in their intricate spatial relationships that are challenging for CNNs to model effectively. To that end, the thigh consists of various different tissue compartments (e.g., flexor/extensor, intermuscular fat) in a relatively small cross-sectional area, where even slight misclassifications can potentially compromise the clinical utility of segmentation algorithms, e.g., in longitudinal monitoring of disease biomarkers. ViTs have demonstrated superior performance in image segmentation compared to CNNs, including segmentation of brain MRI [29]. These architectural differences likely also explain the lower RMSE observed for DINOv2 in our study, compared to MobileNetV2.

Limitations include the modest number of training data for the developed models, based on a prospective study that was acquired on a single MRI scanner type. The lack of external validation limits the interpretability of the proposed framework regarding clinical applicability. Second, the dependence on qualitative gradings by two radiologists may introduce variability. Third, there was a class imbalance in our dataset, which was skewed towards high-quality segmentations (Likert = 5). This could impact the robustness of both the Likert predictions and the regression-based metric signature, particularly for lower-quality segmentations (Likert = 1–3). To address class imbalance, we employed the “class-weight” parameter, which adjusts the loss function by assigning inverse class-frequency weights. The model is therefore compelled to prioritize learning minority classes to minimize overall loss. Future studies should expand this dataset with a more balanced distribution of Likert scores, and with data acquired from different institutions, scanners and patient populations. Fourth, the manual segmentations used as reference standards were limited to only three axial slices per leg. Therefore, the reference data may not fully represent the anatomical variability across the entire thigh, potentially impacting the generalizability of the performance metrics. Furthermore, no true volumetric 3D ground truth was available. The reported performance metrics are therefore 2D and slice-based, relying on sparse 2D manual annotations as a proxy for 3D anatomy, and do not capture interslice continuity or true volumetric accuracy. This distinction is particularly important for the reported performance of the weighted metric combination, including Hausdorff distance, which behaves differently in 2D versus 3D analyses. Moreover, manual segmentations were only performed by a single reader, without inter-observer reliability analysis. However, the primary objective of our study was not to establish a ground truth segmentation benchmark nor to quantify segmentation reproducibility per se. Rather, the aim was to evaluate radiologists’ subjective satisfaction with segmentation maps, and to investigate how conventional similarity metrics relate to perceived clinical acceptability. Therefore, potential segmentation variability would not confound the principal research question, but instead reflect real-world annotation uncertainty that clinicians encounter in practice. Fifth, our model is based on task-specific metric weights and only applies to the fat fraction map of the Dixon sequence, excluding the other Dixon image contrasts. However, the fat fraction maps yield important quantitative information for tissue characterization, serving as potential biomarkers in body composition profiling [30]. Moreover, the thigh is an anatomic region that generally provides favorable imaging conditions and is often used as an efficient alternative to whole-body MRI. Future work could expand the dataset with integration of additional MRI contrasts to further enhance model generalizability and clinical applicability. As the proposed framework is inherently adaptable, we envision that it could be extended to other anatomical regions or different MRI sequences by recalibrating the weighted metric model with qualitative scores specific to the region and/or task and by refining the optimal metric weights.

In conclusion, our proposed framework provides a tailored, weighted multiparametric evaluation of MRI segmentation performance, facilitating the development of more clinically relevant and reliable DL-automated tools. By combining quantitative metrics with qualitative user feedback, this approach can aid in improving the translation of segmentation algorithms from research to clinical practice.

## Figures and Tables

**Table 1:** Subject characteristics. Continuous variables are provided in the format: mean (SD).

Characteristic	Value
*N*	68
Age, yrs	29.4 (5.6)
Female, *n* (%)	31 (45%)
Male, *n* (%)	37 (55%)
Height, cm	173.5 (8.7)
Weight, kg	77.5 (10.2)
BMI, kg/m^2^	25.7 (2.2)

**Table 2:** Classification results of feature extractors MobileNetV2 and DINOv2.

	Training Set				Test Set			
Likert/Metric	Precision (95% CI)	Recall (95% CI)	F1-Score (95% CI)	Support	Precision (95% CI)	Recall (95% CI)	F1-Score (95% CI)	Support
MobileNetV2								
Likert 2	0.00 (0.000, 0.000)	0.00 (0.000, 0.000)	0.00 (0.000, 0.000)	2	0.00 (0.000, 0.000)	0.00 (0.000, 0.000)	0.00 (0.883, 0.917)	1
Likert 3	0.76 (0.699, 0.821)	0.44 (0.362, 0.518)	0.56 (0.482, 0.638)	155	0.78 (0.713, 0.847)	0.36 (0.209, 0.511)	0.49 (0.333, 0.647)	39
Likert 4	0.69 (0.667, 0.713)	0.64 (0.616, 0.664)	0.67 (0.647, 0.693)	1546	0.60 (0.551, 0.649)	0.56 (0.510, 0.610)	0.58 (0.531, 0.629)	386
Likert 5	0.89 (0.881, 0.899)	0.93 (0.923, 0.937)	0.91 (0.902, 0.918)	4823	0.87 (0.858, 0.882)	0.90 (0.883, 0.917)	0.88 (0.862, 0.898)	1206
Accuracy			0.85 (0.821–0.879)	6526			0.81 (0.748–0.872)	1632
Macro Avg	0.59	0.50	0.53 (0.450–0.610)	6526	0.56	0.46	0.49 (0.412–0.568)	1632
Weighted Avg	0.84	0.85	0.84 (0.811–0.869)	6526	0.80	0.81	0.80 (0.737–0.863)	1632
DINOv2								
Likert 2	0.000 (0.000, 0.000)	0.000 (0.000, 0.000)	0.000 (0.000, 0.000)	1	0.000 (0.000, 0.000)	0.000 (0.000, 0.000)	0.000 (0.000, 0.000)	2
Likert 3	0.667 (0.518, 0.816)	0.256 (0.119, 0.393)	0.370 (0.218, 0.522)	39	0.849 (0.805, 0.893)	0.290 (0.219, 0.361)	0.433 (0.355, 0.511)	155
Likert 4	0.685 (0.638, 0.732)	0.523 (0.473, 0.573)	0.593 (0.544, 0.642)	386	0.749 (0.727, 0.771)	0.574 (0.549, 0.599)	0.650 (0.626, 0.674)	1546
Likert 5	0.864 (0.850, 0.878)	0.947 (0.934, 0.960)	0.903 (0.886, 0.920)	1206	0.876 (0.869, 0.883)	0.960 (0.954, 0.966)	0.916 (0.908, 0.924)	4823
Accuracy			0.830 (0.771–0.889)	1632			0.853 (0.825–0.881)	6526
Macro Avg	0.554	0.432	0.467 (0.389–0.545)	1632	0.618	0.456	0.500 (0.460–0.540)	6526
Weighted Avg	0.816	0.830	0.817 (0.756–0.878)	1632	0.845	0.853	0.841 (0.812–0.870)	6526

Avg = average.

**Table 3:** Logistic and Lasso regression model selected features and weights.

Metric	Weight
Logistic regression model	
mean_dice_all_classes	−0.027694
mean_iou_all_classes	0.031137
mean_hd_all_classes	−0.000803
dice_c1	0.012618
iou_c1	−0.009002
hd_c1	0.000119
dice_c2	−0.286543
iou_c2	0.288921
hd_c2	0.000125
dice_c3	−0.017134
iou_c3	0.011545
hd_c3	0.000127
dice_c4	0.040503
iou_c4	−0.025643
hd_c4	0.000112
dice_c5	0.105878
iou_c5	−0.099639
hd_c5	0.000196
dice_c6	−0.021485
iou_c6	−0.020638
hd_c6	0.000138
Lasso regression model	
iou_c2	0.628232
dice_c5	0.268339
hd_c4	0.071567
iou_c6	0.056555
mean_iou_all_classes	0.043549
dice_c4	0.015714
hd_c1	0.009738
dice_c1	0.006099
hd_c3	−0.011742
dice_c3	−0.019056
hd_c5	−0.021248
hd_c6	−0.025958
mean_hd_all_classes	−0.037513
dice_c6	−0.072334
iou_c5	−0.275892
dice_c2	−0.573012

Hd, hausdorf distance; IOU, intersection over union.

## Data Availability

The data that support the findings of this study will be made available upon reasonable request.

## Abbreviations

CNN	Convolutional neural network
DL	Deep learning
FF	Fat fraction
IOU	Intersection over union
MAE	Mean absolute error
MSE	Mean squared error
RMSE	Root mean square error
ROC_AUC	Receiver operating characteristic area under curve
ViT	Vision transformer

## References

[1] Zhang Y., Shen Z., Jiao R. (2024). Segment anything model for medical image segmentation: Current applications and future directions. Comput. Biol. Med..

[2] Do T.-D., Nonnenmacher T., Burghardt M., Zschaebitz S., Hajiyianni M., Mai E.K., Raab M.-S., Müller-Tidow C., Kauczor H.-U., Goldschmidt H. (2025). AI-Based 3D-Segmentation Quantifies Sarcopenia in Multiple Myeloma Patients. Diagnostics.

[3] Lind L., Strand R., Michaëlsson K., Ahlström H., Kullberg J. (2020). Voxel-wise study of cohort associations in whole-body MRI: Application in metabolic syndrome and its components. Radiology.

[4] Ziegeler K., Kreutzinger V., Akkaya Z., Joseph G.B., Lynch J.A., Lane N.E., McCulloch C.E., Nevitt M., Link T.M. (2025). Thigh-muscle volume change in response to diet, exercise, and weight-loss in overweight and obese individuals—8-year follow-up data from the Osteoarthritis Initiative. Skelet. Radiol..

[5] Yasutomi M., Saito K., Araki Y., Sugimoto K., Yoshimaru D., Shibukawa S., Ishida M. (2026). MRI-Based Assessment of Etiology-Specific Sarcopenia Phenotypes in Chronic Liver Disease: A Comparative Study of MASH and Viral Hepatitis. Diagnostics.

[6] Xu Y., Quan R., Xu W., Huang Y., Chen X., Liu F. (2024). Advances in medical image segmentation: A comprehensive review of traditional, deep learning and hybrid approaches. Bioengineering.

[7] Kiefer L.S., Winter M., Pappa S., Fischer M., Küstner T., Diallo T.D., Calderón E., Bamberg F., Nikolaou K., Yang B. (2026). Age-and BMI-Dependent Psoas and Gluteus Muscle Mass in 27,805 Participants of the Population-Based German National Cohort (NAKO Gesundheitsstudie): A Deep-Learning 3T MRI Study. Diagnostics.

[8] Graf R., Platzek P., Riedel E.O., Ramschütz C., Starck S., Möller H.K., Atad M., Völzke H., Bülow R., Schmidt C.O. (2026). VIBESegmentator: Full body MRI segmentation for the NAKO and UK Biobank. Eur. Radiol..

[9] Müller D., Soto-Rey I., Kramer F. (2022). Towards a guideline for evaluation metrics in medical image segmentation. BMC Res. Notes.

[10] Nai Y.-H., Teo B.W., Tan N.L., O’Doherty S., Stephenson M.C., Thian Y.L., Chiong E., Reilhac A. (2021). Comparison of metrics for the evaluation of medical segmentations using prostate MRI dataset. Comput. Biol. Med..

[11] Kirimtat A., Krejcar O. A Guide and Mini-Review on the Performance Evaluation Metrics in Binary Segmentation of Magnetic Resonance Images. Proceedings of the International Work-Conference on Bioinformatics and Biomedical Engineering.

[12] Derron N., Güntner A.T., Weber I.C., Braun J., Koska İ.Ö., Othman A., Mönch L., von Eckardstein A., Puhan M.A., Beuschlein F. (2025). Alternate-day fasting elicits larger changes in fat mass than time-restricted eating in adults without obesity–A randomized clinical trial. Clin. Nutr..

[13] Huber F.A., Chaitanya K., Gross N., Chinnareddy S.R., Gross F., Konukoglu E., Guggenberger R. (2022). Whole-body composition profiling using a deep learning algorithm: Influence of different acquisition parameters on algorithm performance and robustness. Investig. Radiol..

[14] Ronneberger O., Fischer P., Brox T. U-net: Convolutional networks for biomedical image segmentation. Proceedings of the International Conference on Medical Image Computing and Computer-Assisted Intervention.

[15] Sandler M., Howard A., Zhu M., Zhmoginov A., Chen L.-C. Mobilenetv2: Inverted residuals and linear bottlenecks. Proceedings of the IEEE Conference on Computer Vision and Pattern Recognition.

[16] Oquab M., Darcet T., Moutakanni T., Vo H., Szafraniec M., Khalidov V., Fernandez P., Haziza D., Massa F., El-Nouby A. (2023). Dinov2: Learning robust visual features without supervision. arXiv.

[17] Prion S., Haerling K.A. (2014). Making sense of methods and measurement: Spearman-rho ranked-order correlation coefficient. Clin. Simul. Nurs..

[18] Ding J., Cao P., Chang H.-C., Gao Y., Chan S.H.S., Vardhanabhuti V. (2020). Deep learning-based thigh muscle segmentation for reproducible fat fraction quantification using fat–water decomposition MRI. Insights Imaging.

[19] Agosti A., Shaqiri E., Paoletti M., Solazzo F., Bergsland N., Colelli G., Savini G., Muzic S.I., Santini F., Deligianni X. (2022). Deep learning for automatic segmentation of thigh and leg muscles. Magn. Reson. Mater. Phys. Biol. Med..

[20] Gaj S., Eck B.L., Xie D., Lartey R., Lo C., Zaylor W., Yang M., Nakamura K., Winalski C.S., Spindler K.P. (2023). Deep learning-based automatic pipeline for quantitative assessment of thigh muscle morphology and fatty infiltration. Magn. Reson. Med..

[21] Keles E., Irmakci I., Bagci U. (2022). Musculoskeletal MR image segmentation with artificial intelligence. Adv. Clin. Radiol..

[22] Renard F., Guedria S., Palma N.D., Vuillerme N. (2020). Variability and reproducibility in deep learning for medical image segmentation. Sci. Rep..

[23] Niessen W.J., Bouma C.J., Vincken K.L., Viergever M.A. (2000). Error metrics for quantitative evaluation of medical image segmentation. Performance Characterization in Computer Vision.

[24] Reinke A., Tizabi M.D., Sudre C.H., Eisenmann M., Rädsch T., Baumgartner M., Acion L., Antonelli M., Arbel T., Bakas S. (2021). Common limitations of image processing metrics: A picture story. arXiv.

[25] Maier-Hein L., Eisenmann M., Reinke A., Onogur S., Stankovic M., Scholz P., Arbel T., Bogunovic H., Bradley A.P., Carass A. (2018). Why rankings of biomedical image analysis competitions should be interpreted with care. Nat. Commun..

[26] Seghier M.L. (2024). Image Segmentation Evaluation With the Dice Index: Methodological Issues. Int. J. Imaging Syst. Technol..

[27] Taha A.A., Hanbury A. (2015). Metrics for evaluating 3D medical image segmentation: Analysis, selection, and tool. BMC Med. Imaging.

[28] Müller-Franzes G., Khader F., Siepmann R., Han T., Kather J.N., Nebelung S., Truhn D. (2025). Medical slice transformer for improved diagnosis and explainability on 3D medical images with DINOv2. Sci. Rep..

[29] Mittal P., Sharma B., Yadav D. Comparative Analysis between CNN and ViT using Brain MRI Dataset. Proceedings of the 2024 Eighth International Conference on Parallel, Distributed and Grid Computing (PDGC).

[30] Reeder S.B., Hu H.H., Sirlin C.B. (2012). Proton density fat-fraction: A standardized MR-based biomarker of tissue fat concentration. J. Magn. Reson. Imaging JMRI.

